# Iron quantification in basal ganglia using quantitative susceptibility mapping in a patient with ALS: a case report and literature review

**DOI:** 10.3389/fnins.2023.1229082

**Published:** 2023-09-27

**Authors:** Sadegh Ghaderi, Seyed Amir Hossein Batouli, Sana Mohammadi, Farzad Fatehi

**Affiliations:** ^1^Department of Neuroscience and Addiction Studies, School of Advanced Technologies in Medicine, Tehran University of Medical Sciences, Tehran, Iran; ^2^Neuromuscular Research Center, Department of Neurology, Shariati Hospital, Tehran University of Medical Sciences, Tehran, Iran; ^3^Department of Medical Sciences, School of Medicine, Iran University of Medical Sciences, Tehran, Iran

**Keywords:** MRI, QSM, ALS, basal ganglia, iron

## Abstract

**Background:**

Quantitative susceptibility mapping (QSM) is a magnetic resonance imaging (MRI) technique that can measure the magnetic susceptibility of tissues, which can reflect their iron content. QSM has been used to detect iron accumulation in cortical and subcortical brain regions. However, its application in subcortical regions such as the basal ganglia, particularly the putamen, is rare in patients with amyotrophic lateral sclerosis (ALS).

**Case presentation and literature review:**

We present the case of a 40-year-old male patient with ALS who underwent an MRI for QSM. We compared his QSM images with those of a control subject and performed a quantitative analysis of the magnetic susceptibility values in the putamen regions. We also reviewed the literature on previous QSM studies in ALS and summarized their methods and findings. Our QSM analysis revealed increased magnetic susceptibility values in the bilateral putamen of the ALS patient compared to controls, indicating iron overload. This finding is consistent with previous studies reporting iron dysregulation in subcortical nuclei in ALS. We also discussed the QSM processing techniques used in our study and in the literature, highlighting their advantages and limitations.

**Conclusion:**

This case report demonstrates the potential of QSM as a sensitive MRI biomarker for evaluating iron levels in subcortical regions of ALS patients. QSM can provide quantitative information on iron deposition patterns in both motor and extra-motor areas of ALS patients, which may help understand the pathophysiology of ALS and monitor disease progression. Further studies with larger samples are needed to validate these results and explore the clinical implications of QSM in ALS.

## Introduction

Amyotrophic lateral sclerosis (ALS) is a devastating neurodegenerative disorder that affects both upper and lower motor neurons (UMN and LMN), leading to progressive muscle weakness, paralysis, and death (Bhattarai et al., 2022; Ghaderi et al., 2023a; Marshall et al., 2023; Mohammadi and Ghaderi, 2023). ALS is affected by motor and extra-motor neurodegeneration (Ragagnin et al., 2019; Rojas et al., 2020; Reyes-Leiva et al., 2022). The neuropathological mechanisms underlying ALS involve complex interactions between genetic, environmental, and cellular factors, resulting in motor neuron vulnerability and neuroinflammation (Mejzini et al., 2019; Le Gall et al., 2020; Keon et al., 2021). Accumulating evidence suggests that iron dysregulation and deposition can play a crucial role in the pathogenesis of ALS, contributing to oxidative stress and neuronal damage (Kupershmidt and Youdim, 2023; Long et al., 2023). Iron is an essential element for cellular metabolism, but excess iron can generate reactive oxygen species (ROS) that damage cellular components, such as lipids, proteins, and DNA (Ying et al., 2021). Therefore, iron homeostasis is tightly regulated in the brain by various proteins, such as transferrin, ferritin, and hepcidin (Singh et al., 2014).

Iron dysregulation and deposition have a variety of effects on neuronal function and survival. Iron, for example, could change the expression and activity of glutamate receptors and transporters, resulting in excitotoxicity and synaptic dysfunction. Iron can trigger mitochondrial dysfunction, which reduces energy production and increases ROS production (Cheng et al., 2022). In addition to stimulating microglia and astrocytes into action, iron may stimulate neuroinflammation and cytokine release. Furthermore, iron can react with other metals such as copper and zinc, affecting their availability and toxicity. In addition, the misfolded proteins superoxide dismutase 1 (SOD1) and TAR DNA-binding protein 43 (TDP-43), which are linked to both familial and sporadic ALS, may be aggregated and cleared by iron (Basso et al., 2013; Ndayisaba et al., 2019).

Magnetic resonance imaging (MRI) is a powerful tool for diagnosing various medical conditions, such as neurological disorders (Kollewe et al., 2012; Bhattarai et al., 2022; Ghaderi, 2023; Ghaderi et al., 2023b; Mohammadi et al., 2023). Quantitative susceptibility mapping (QSM) is a sensitive MRI technique for detecting magnetic susceptibility changes in tissues (Acosta-Cabronero et al., 2018). QSM is a technique that can be used in conjunction with MRI to measure the magnetic susceptibility of tissues, which reflects how easily a tissue becomes magnetized in a magnetic field (Ravanfar et al., 2021). Magnetic susceptibility measures how easily a tissue becomes magnetized in a magnetic field (Conte et al., 2021). Tissues with high magnetic susceptibility, such as iron-rich tissues, can distort the magnetic field in MRI scans (Duyn, 2013). QSM can provide accurate and reliable estimates of iron concentration in various brain regions, such as the cortex, basal ganglia, and cerebellum, and QSM has shown promising results in detecting iron deposition in neurodegenerative diseases, including ALS (Ravanfar et al., 2021).

Susceptibility-weighted imaging (SWI) is another MRI technique that can visualize tissues with high magnetic susceptibility (Liu et al., 2021). SWI combines magnitude and phase information to qualitatively display tissue magnetic field variations, but it is affected by regional field effects and image artifacts that vary with image parameters (Haacke et al., 2009; Mittal et al., 2009; Haller et al., 2021). SWI has also been used to diagnose and monitor conditions that involve iron deposition, such as neurodegenerative diseases and neuromuscular disorders (Schweitzer et al., 2015; Lee et al., 2017; Welton et al., 2019), but it has lower diagnostic performance than QSM (Liu et al., 2015; Adams et al., 2017). Furthermore, SWI is affected by regional field effects and image artifacts that vary with image parameters, and it only provides qualitative information on tissue magnetic susceptibility (Liu et al., 2015).

Numerous studies have used QSM and SWI to investigate iron deposition in the motor cortex of ALS patients, which is the primary site of UMN degeneration. These studies have consistently reported increased iron accumulation in the motor cortex in ALS patients compared to healthy controls, suggesting a link between cortical iron dysregulation and neuronal loss (Schweitzer et al., 2015; Costagli et al., 2016, 2022; Lee et al., 2017). Subcortical structures, such as the basal ganglia, thalamus, and brainstem, are also rich in iron and vulnerable to oxidative stress (Péran et al., 2009; Lee and Lee, 2019). The putamen and globus pallidus (GP) were chosen as the primary components of the basal ganglia, which are involved in motor control, movement disorders, and cognitive processes (Macpherson and Hikida, 2019; Saad et al., 2020). Because the basal ganglia are iron-rich and susceptible to oxidative stress, they are prospective targets for ALS susceptibility alterations (Ward et al., 2014). Previous research using QSM showed more iron deposition in the putamen and GP of ALS patients, but they did not focus on sensitivity levels for precise quantification of these regions, especially the putamen, which would increase its significance (Acosta-Cabronero et al., 2018; Ravanfar et al., 2021). As a consequence, we planned to use QSM to offer a quantitative investigation of iron deposition in the putamen and GP of an ALS patient and compare it to healthy controls. We did not include additional motor-related subcortical regions in the study, such as the subthalamic nucleus (STN), substantia nigra (SN), and red nucleus (RN) since previous research has revealed that putamen and GP are more vulnerable to iron buildup and dysregulation (Acosta-Cabronero et al., 2018; Ravanfar et al., 2021). Moreover, subcortical iron abnormalities may have clinical implications for ALS patients as they may be associated with cognitive impairment, behavioral changes, and extrapyramidal symptoms (Crockford et al., 2018; Urso et al., 2022). However, the role of iron deposition in subcortical structures implicated in ALS remains poorly understood, with limited studies focusing on these regions. Investigating subcortical iron deposition could provide valuable insights into the neuropathological mechanisms underlying ALS and improve our understanding of disease progression and potential therapeutic targets.

The basal ganglia comprise a group of interconnected subcortical nuclei involved in motor control, movement disorders, and cognitive functions (Urso et al., 2022). Given the integral role of the basal ganglia in motor functions and control, examining whether abnormal iron distribution occurs in the basal ganglia of ALS patients is crucial to gain further insights into the pathogenesis of ALS (Castelnovo et al., 2023). In light of the current knowledge gaps and the potential value of studying susceptibility abnormalities in subcortical structures in ALS, we undertook a single case–control study using QSM to investigate iron quantification in the basal ganglia of a patient with ALS. Finally, this case report and literature review aimed to explore the association between susceptibility abnormalities and the neuropathological mechanisms underlying ALS, discuss the uncertainties in this research field, and contribute to the growing body of evidence on subcortical iron deposition in ALS.

## Case presentation

A 40-year-old male patient presented with a 3-year history of ALS. The patient exhibited diffuse muscle atrophy, fasciculations, weakness, and hyperreflexia. Both UMN and LMN symptoms were observed, and the patient had an ALS Functional Rating Scale-Revised (ALSFRS-R) score of 38. Notably, there was no evidence of cognitive or behavioral disorders. The control subject was a 40-year-old male without any past medical history of neurodegeneration, neuromuscular disorders, or trauma.

## Imaging and QSM analysis

**Figure 1:**
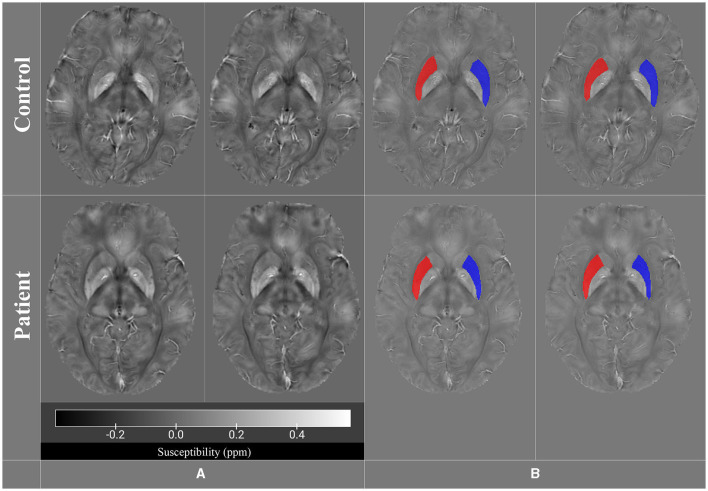
QSM images for both control and patient groups in **(A)**, while **(B)** displays the putamen region of interest (ROI) in all four images (control and patient).

To address these inquiries, QSM analyses were performed. Accordingly, we acquired the MRI images using a Siemens 3.0 Tesla scanner (Prisma, 2016) with a superconductive zero helium boil-off 3T magnet at the National Brain Mapping Laboratory (NBML, Tehran, Iran). The imaging parameters were as follows: TR: 41 ms, TE: 21 ms, flip angle: 15°, slice thickness: 2 mm, voxel resolution: 0.6 × 0.6 × 2.0 mm, ETL: 4, pixel spacing: 0.625/0.625 mm, FoV read: 240 mm, FoV phase: 81.3%, matrix size: 384 × 312, slice per slab =: 72, slice resolution = 100%, PAT mode = GRAPPA, and coil: 64-channel head/neck coil. The study was approved by the Ethics Committee of Tehran University of Medical Sciences (Ethical Code: IR.TUMS.MEDICINE.REC.1400.1173). The QSM was reconstructed from the SWI sequence data acquisition using the following steps based on methodological studies: (1) phase unwrapping using the Laplacian-based method (Li et al., 2014, 2022; Acosta-Cabronero et al., 2018); (2) background field removal using the V-SHARP method (Fang et al., 2017; Kan et al., 2018; Wang et al., 2020; Li et al., 2022); and (3) field-to-susceptibility inversion using streaking artifacts reduction QSM (STAR-QSM) method (Wei et al., 2015; Wang et al., 2020; Conte et al., 2021; Yaghmaie et al., 2021). QSM calculations were performed using MATLAB, STI Suite (a MATLAB toolbox) (Li et al., 2014). The reconstructed QSM images were analyzed to provide information about the magnetic properties of the tissue, such as iron accumulations. MRIcroGL was used to extract and export two axial slices from the control and patient QSM images (Uddin et al., 2022). The selected images focused on studying the basal ganglia, especially the putamen. We manually defined the region of interest (ROI) for the putamen by a neurologist using ITK-SNAP software. ITK-SNAP was employed to define ROIs and calculate the number of voxels, volumes, and mean intensity values for the putamen regions (Yushkevich et al., 2016) (Figure 1).

**Figure 2:**
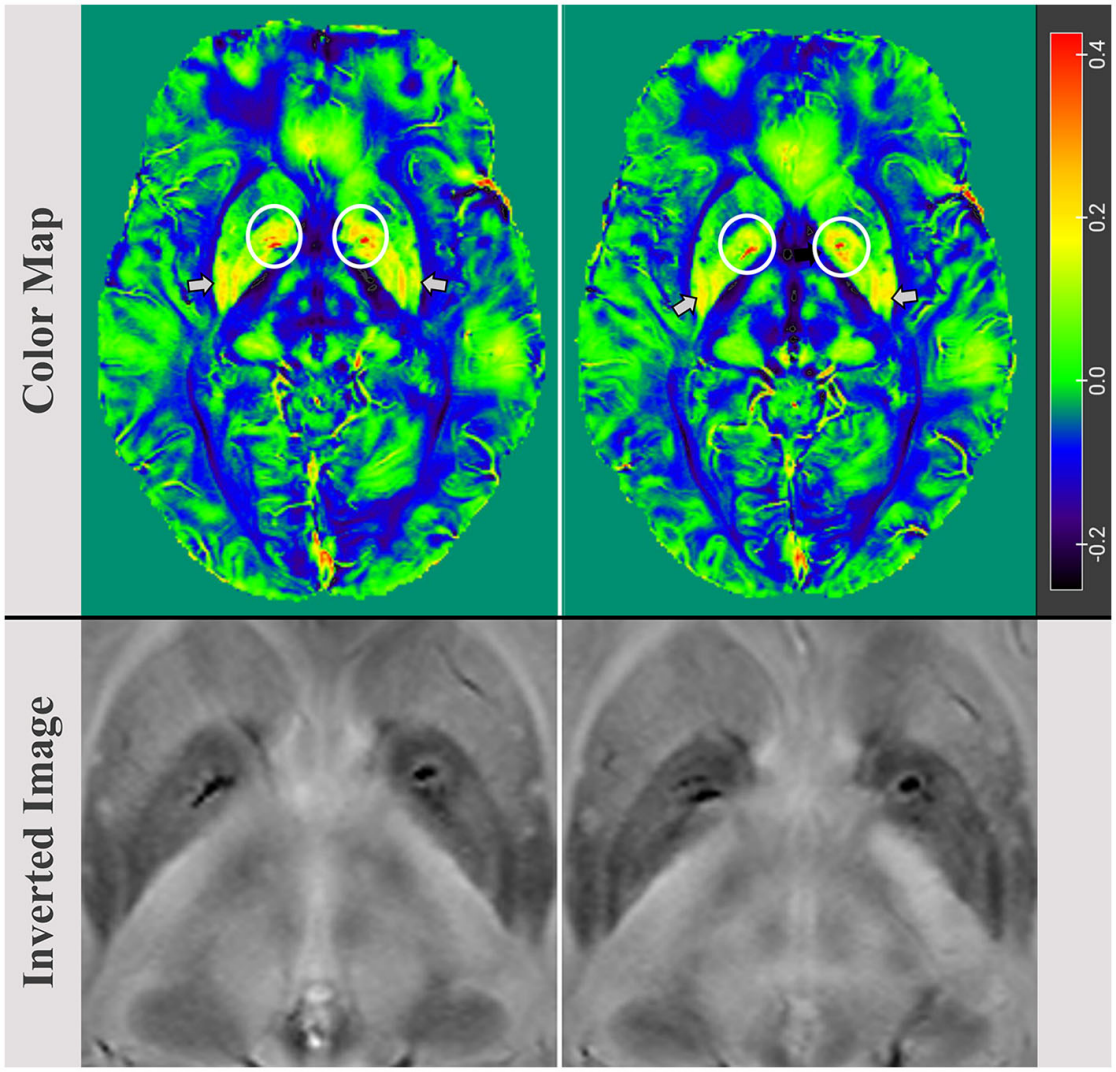
Color map images of the bilaterally basal ganglia regions, with an arrow indicating an increase in magnetic susceptibility in the putamen and a circle highlighting iron load accumulation in the globus pallidus (GP). The maximum values in the left and right GP were 0.086 (ppm) and 0.079 (ppm), respectively. Inverted images reveal and confirm bilaterally localized iron accumulations in the basal ganglia, particularly in the GP.

MRIcroGL was also used to generate color maps of the bilateral basal ganglia region, with arrows indicating increased magnetic susceptibility in the putamen and circles highlighting high iron accumulation in the GP. Finally, MRIcro was utilized to export inverted images displaying the basal ganglia region and high local iron load in the bilateral GP (Figure 2).

## Results and discussion

Table 1 presents the number of voxels, volumes, and mean intensity values for the left and right putamen in both control and patient images. The study quantified iron content in the putamen region of control and patient images. Results show a higher iron content in patient images for both left (0.068 ± 0.008) and right (0.062 ± 0.007) putamen regions. The control left putamen region has 1,518 voxels with a volume of 1,185.9 mm^3^ and an image mean ± SD of 0.034 ± 0.005. Similarly, the control right putamen region has 1,682 voxels with a volume of 1,314.1 mm^3^ and image mean ± SD of 0.041 ± 0.005. The patient's left putamen region has 1,448 voxels with a volume of 1,131.3 mm^3^, while the patient's right putamen region has 1,318 voxels with a volume of 1,029.7 mm^3^.

**Table 1:** Iron quantification in the putamen.

**Slice**	**Region**	**Number of voxels**	**Volume (mm^3^)**	**Image mean ± SD**
**Control images**				
1	Left putamen	1,518	1,185.9	0.034 ± 0.005
2	Right putamen	1,682	1,314.1	0.041 ± 0.005
**Patient images**				
1	Left putamen	1,448	1,131.3	0.068 ± 0.008
2	Right putamen	1,318	1,029.7	0.062 ± 0.007

Our review of the literature showed that most previous QSM studies in ALS and other neurodegenerative diseases focused on iron changes in the motor cortex and corticospinal tract. Table 2 is a key source of information on past studies that have employed QSM in the examination of motor neuron diseases (MNDs), particularly ALS. However, a few reported quantitative QSM values in subcortical regions such as the basal ganglia, especially the putamen. The quantitative analysis performed in this study is valuable because it provides objective measurements of magnetic susceptibility values in the basal ganglia of an ALS patient, which can be compared with normal controls and other brain regions. This analysis can also reveal the extent and distribution of iron accumulation in these regions, which may reflect different pathological processes or disease stages. The quantitative analysis performed in this study is unique because, to the best of our knowledge, no previous study has reported simultaneous specific qualitative and quantitative QSM data in the putamen of an ALS patient. Quantifying qualitative data such as we did is very useful to reduce human error (Wooldridge et al., 2018). Prior research on neurodegenerative diseases has primarily presented qualitative SWI data along with some quantitative QSM values in certain cortical regions, as well as indications in the putamen and GP. Our study offers new and valuable insights into the magnetic susceptibility of the basal ganglia, particularly the putamen, in relation to ALS.

**Table 2:** Summary of quantitative susceptibility mapping studies in ALS.

**Records**	**Patients**	**MRI field and coils/techniques**	**QSM**	**Region(s)**	**Finding**
Schweitzer et al. (2015)	12 ALS and 4 PLS 23	3 T/T2^*^-w and QSM	MEDI	Motor cortex	• ↑ Magnetic susceptibility (χ) in ALS and PLS compared to controls • ↑ Sensitivity and specificity for QSM in diagnosis of ALS and PLS compared to controls
Costagli et al. (2016)	17 ALS 13	7 T (32-channel coil)/3D ME-GRE T2^*^-w, 2D GRE, and QSM	Laplacian-based V-SHARP iLSQR	M1	• ↑ Magnetic susceptibility In ALS patients, magnetic susceptibility is observed in the middle and deep layers of the M1, where it co-localizes with T2^*^ hypointensity
Lee et al. (2017)	26 26	3 T/T2-w and QSM	HARPERELLA HARPERELLA N/A	Motor cortex and subcortical WM	• ALS patients ↓ subcortical WM mean compared to healthy controls ALS patients: cortexmax ↔ ALSFRS-R scores (negative correlation)
Acosta-Cabronero et al. (2018)	28 39	3 T (32-channel coil)/QSM, T1-w MP-RAGE, 3D SPGR, and DTI	Laplacian-based SHARP MEDI	Motor cortex, CST, and a range of subcortical nuclei	ALS patients exhibit: • ↑χ in the motor cortex (precentral gyrus), SN, GP, RN, PUT, HP, and pars opercularis on the right side • ↓χ in the CST • Higher χ in the SN and GP in bulbar-onset compared to limb-onset ALS patients • There is no correlation between ALSFRS scores and χ in any of the investigated regions Correlation analysis between QSM and DTI revealed: • Positive correlation between χ and fractional anisotropy • Negative correlation between χ and mean diffusivity and radial diffusivity
Weidman et al. (2019)	38 ALS and 5 PLS 15	3 T (8-channel coil)/QSM, T2, 3D T2 FLAIR, and 3D MP-RAGE	N/A	Motor cortex and central sulcus	• UMN group ↑ QSM MMCS compared to the non-UMN group QSM MMCS cutoff value = 65.6; sensitivity = 30%; specificity = 100% • ALS/PLS patients ↑ QSM MMCS compared to mimics
Donatelli et al. (2019)	36 ALS and 19 ALS-bulbar N/A	3 T (8-channel coil)/QSM and 3D ME-GRE T2^*^-w	Laplacian-based V-SHARP iLSQR	M1 (orofacial region)	• Magnetic susceptibility map ↓↑ among different visual scores in M1 ALS patients with bulbar symptoms ↑ T2^*^ hypointensities in orofacial M1 • T2^*^ hypointensities → ↑ magnetic susceptibility in QSM in same region
Welton et al. (2019)	21 63	3 T (32-channel coil)/QSM, DKI, and 3D ME-GRE T2^*^-w	MEDI	Motor cortex	• ALS patients ↔ healthy controls: ↓ diffusion kurtosis metrics and ↑ iron concentration in the motor cortex Iron deposition and diffusion kurtosis metrics → highest diagnostic accuracy
Contarino et al. (2020)	42 23	3 T/QSM, 3D T1-w, and 3D T2-w SPGR	N/A V-SHARP STAR	Precentral cortex and motor cortex	• Susceptibility skewness → the biomarker of UMN impairment in ALS patients
Bhattarai et al. (2020)	13 11	3 T (32-channel coil)/QSM, T1- w MP-RAGE, and T2^*^-w	Laplacian-based V-SHARP iLSQR	M1 and premotor area	• Limb-onset ALS patients ↑ QSM values in left posterior M1 and right anterior M1 compared to control participants Lumbar onset ALS patients ↑ QSM values in primary motor and pre-motor areas compared to control subjects • Lumbar onset ALS patients ↑ iron dysregulation in the motor cortex compared to cervical onset ALS patients • Limb-onset ALS patients ↑ iron concentration in M1 compared to control participants
Wang et al. (2020)	9 3	7 T (32-channel coil)/QSM and 3D GRE	PRELUDE V-SHARP, PDF, and LBV STAR, MEDI, and TKD	M1, anterior cingulate, and visual cortices	• Susceptibility and R2* maps → quantitative imaging marker for ALS diagnosis ALS brains ↑ susceptibility and R2* values in M1 compared to control brains • Susceptibility and R2^*^↔ myelin and ferritin estimates (positive correlation) • Four out of nine ALS brains ↑ susceptibility and R2* values in M1 (visible hyperintensity)
Conte et al. (2021)	47 23	3 T/QSM, 3D T1-w, 3D FLAIR, and T2-w	N/A V-SHARP STAR	Precentral cortex	• Susceptibility in precentral cortex ↔ UMN or LMN prevalence in ALS phenotypes UMN-ALS patients ↑ susceptibility in the precentral cortex compared to Np-ALS and LMN-ALS patients
Dean et al. (2021)	5 35	3 T/QSM, T2-w, 3D T1-w MP-RAGE, and T2-w FLAIR	N/A	Motor cortex	• ALS patients ↑ magnetic susceptibility values in hand and face homunculi compared to normal population
Canna et al. (2021)	33 28	3 T (32-channel coil)/QSM, T1-w, FLAIR, 3D ME, and 3D-PCASL	Laplacian-based V-SHARP Dipole Convolution	Precentral cortex and subcortical structures	• ALS patients ↑ QSM in precentral gyrus compared to healthy controls QSM in left precentral gyrus ↔ ALSFRS-R scores in ALS patients (significant correlation) • QSM in right precentral gyrus ↔ disease durations in ALS patients (significant correlation) • QSM + CBF + GM volumetry → effective differentiation between ALS and PLS phenotypes in the motor cortex and between ALS and healthy controls in cortical and subcortical regions
Li et al. (2022)	34 34	3 T (8-channel coil)/QSM, 3D T1-w and 3D GRE	Laplacian-based V-SHARP STAR	Precentral gyrus and thalamus	• ALS patients ↑ iron accumulation in the motor cortex and thalamus; iron accumulation ↔ GM atrophy and disease severity ALS patients ↓ GM volume in the bilateral thalamus and precentral gyrus • ALS patients: ↑ QSM values ↔↓ GM volume (association) • ALS patients: iron accumulation in the left precentral gyrus ↔ UMN score (positive correlation); GM volume in the left precentral gyrus ↔ UMN score (negative correlation) • ALS patients: thalamic iron deposition ↔ ALSFRS-R score (negative association)

2D, two dimensional; 3D, three dimensional; ALS, amyotrophic lateral sclerosis; ALSFRS-R, ALS functional rating scale-revised; CBF, cerebral blood flow; CST, corticospinal tract; DKI, diffusion kurtosis imaging; DTI, diffusion tensor imaging; FLAIR, fluid-attenuated inversion recovery; GM, gray matter; GP, globus pallidus; HARPERELLA, Harmonic Phase Removal Using the Laplacian Operator; iLSQR, improved sparse linear equation and least-squares; LBV, Laplacian boundary value; LMN, lower motor neuron; M1, primary motor cortex; ME-GRE, multi-echo gradient echo; MEDI, morphology enabled dipole inversion; MMCS, maximum motor cortex susceptibility; MRI, magnetic resonance imaging; MP-RAGE, magnetization-prepared rapid acquisition with gradient echo; Np-ALS, No clinically ALS; PDF, probability density function; PLS, primary lateral sclerosis; PRELUDE, phase region expanding labeler for unwrapping discrete estimates; PUT, putamen; QSM, quantitative susceptibility mapping; RN, red nucleus; SPGR, spoiled gradient echo; STAR, sparsity constrained tissue adaptive regularization; T2^*^-w, T2^*^-weighted; TKD, truncated k-space division; UMN, upper motor neuron; V-SHARP, variable-kernel sophisticated harmonic artifact reduction for phase data; WM, white matter.

Magnetic susceptibility (χ) measures how easily a material can become magnetized under the influence of an external magnetic field.

Symbols: ↑: means “increase” or “elevated”; ↓: means “decrease” or “reduced”; ← or → : means “leads to”, “results in”; ↓↑: means “fluctuation” or “variation”; ↔: means “bidirectional” or “two-way”.

Our QSM analysis found that the ALS patient exhibited higher magnetic susceptibility values in the bilateral putamen compared to controls, indicating increased iron accumulation. Several studies have reported increased magnetic susceptibility in the motor cortex and other regions, which could be indicative of iron accumulation (Schweitzer et al., 2015; Costagli et al., 2016; Acosta-Cabronero et al., 2018; Donatelli et al., 2019; Bhattarai et al., 2020; Li et al., 2022). Our results are consistent with these findings, providing further evidence for the potential role of iron dysregulation in the pathophysiology of ALS. Our finding is consistent with previous studies that reported iron overload in the putamen and other subcortical nuclei in ALS patients and suggests QSM can serve as a valuable imaging biomarker for evaluating iron deposition (Acosta-Cabronero et al., 2018; Li et al., 2022). Excess iron in these regions can promote oxidative stress and neuroinflammation and impair normal cellular functions such as axonal transport and myelination, which may contribute to neurodegeneration in ALS (Ward et al., 2014; Ndayisaba et al., 2019).

Multiple QSM studies have reported increased magnetic susceptibility in ALS patients compared to controls, indicating excessive iron accumulation. Specifically, in these studies, magnetic susceptibility was increased in the motor cortex, subcortical white matter (WM), and basal ganglia (especially the putamen, substantia nigra, and GP) (Schweitzer et al., 2015; Costagli et al., 2016; Acosta-Cabronero et al., 2018; Donatelli et al., 2019; Bhattarai et al., 2020; Li et al., 2022). In one study, higher pallidal magnetic susceptibility was observed in bulbar-onset vs. limb-onset ALS patients (Acosta-Cabronero et al., 2018). Another study based on limb-onset ALS patients displayed elevated QSM values in the left posterior M1 and right anterior M1 compared to controls (Bhattarai et al., 2020) and limb-onset ALS patients exhibited higher iron concentrations in M1 than controls (Bhattarai et al., 2020). The previous literature is rare, but consistent with our results, which showed increased magnetic susceptibility in multiple structures cortical and subcortical, especially in the putamen and GP.

Considering the previous evidence in this area, the UMN group displayed greater QSM mean magnetic susceptibility contrast in the motor cortex compared to the non-UMN group (Weidman et al., 2019). A cutoff value of 65.6 for quantitative susceptibility mapping and mean magnetic susceptibility contrast showed 30% sensitivity and 100% specificity for differentiating ALS/primary lateral sclerosis (PLS) from mimics (Weidman et al., 2019). Magnetic susceptibility was heterogeneous among different visual scores of iron deposition in the primary motor cortex (M1) (Donatelli et al., 2019). These previous findings, which are in line with our findings, indicate that magnetic susceptibility can be utilized to distinguish and identify the disease and its various stages.

We can refer to the findings of these studies regarding the alterations of specific biomarkers including susceptibility and R2^*^ maps, T2^*^ hypointensity, and susceptibility skewness. In one study, susceptibility and R2^*^ maps were suggested as quantitative imaging markers for ALS diagnosis (Wang et al., 2020). Susceptibility in the precentral cortex did not correlate with UMN or LMN prevalence in ALS phenotypes but was higher in UMN-ALS patients compared to neuropsychologically normal ALS and LMN-ALS patients (Canna et al., 2021; Conte et al., 2021; Li et al., 2022). ALS patients with bulbar symptoms exhibited greater T2^*^ hypointensities in the orofacial M1 (Acosta-Cabronero et al., 2018; Donatelli et al., 2019). T2^*^ hypointensities were correlated with increased magnetic susceptibility in the same region. Compared to controls, ALS patients showed decreased diffusion kurtosis metrics and increased iron concentration in the motor cortex (Welton et al., 2019). Iron deposition and diffusion kurtosis metrics demonstrated the highest diagnostic accuracy. Susceptibility skewness was proposed as a biomarker of UMN impairment in ALS (Contarino et al., 2020). Similar to T2^*^ hypointensity, R2^*^ mapping, and susceptibility skewness, as well as other imaging biomarkers including alterations in diffusion metrics, changes in QSM mean magnetic susceptibility can be introduced as a very important biomarker in subcortical nuclei to diagnose elevated iron concentration.

Some studies have reported multiple imaging and cognitive biomarkers. In one study, ALS patients exhibited increased magnetic susceptibility values in the hand and face homunculi compared to normal controls (Dean et al., 2021). In another, QSM in the left precentral gyrus correlated with ALSFRS-R scores, while QSM in the right precentral gyrus correlated with disease duration (Canna et al., 2021). Combined QSM, cerebral blood flow (CBF), and gray matter (GM) volumetry effectively differentiated ALS from PLS in the motor cortex and ALS from controls in cortical and subcortical areas (Canna et al., 2021). In another study in ALS patients, increased iron accumulation in the motor cortex and thalamus was associated with GM atrophy and disease severity (Li et al., 2022). ALS patients showed decreased GM volume in the bilateral thalamus and precentral gyrus. QSM values were associated with decreased GM volume. Iron accumulation in the left precentral gyrus positively correlated with the UMN score, while GM volume in the left precentral gyrus negatively correlated with the UMN score (Li et al., 2022). In sum, by measuring the magnetic susceptibility changes in MND patients with QSM and associating the QSM findings with other imaging outcomes and cognitive biomarkers, we can enhance the quality of the results and increase their rigorousness.

It is important to note that various QSM processing techniques have been employed in the literature, including phase unwrapping, background field removal, and map reconstruction methods (Table 2). Our QSM analysis steps were based on those used in previous clinical studies. These included (1) Laplacian-based phase unwrapping (Costagli et al., 2016; Acosta-Cabronero et al., 2018; Donatelli et al., 2019; Bhattarai et al., 2020; Canna et al., 2021; Li et al., 2022), (2) V-SHARP for background field removal (Costagli et al., 2016; Donatelli et al., 2019; Bhattarai et al., 2020; Contarino et al., 2020; Wang et al., 2020; Canna et al., 2021; Conte et al., 2021; Li et al., 2022), and (3) star QSM for map reconstruction (Contarino et al., 2020; Wang et al., 2020; Conte et al., 2021; Li et al., 2022). These techniques have been widely utilized in recent ALS research, as demonstrated by the studies summarized in the table above, and have proven to be reliable and effective in measuring magnetic susceptibility in various brain regions. These techniques are commonly used and have been shown to provide high-quality QSM maps with minimal streaking artifacts (Wang et al., 2020; Conte et al., 2021; Li et al., 2022). Differences in these techniques may contribute to the variability in the reported findings. Standardization of QSM processing methods could facilitate more reliable comparisons across studies and improve the clinical applicability of QSM in ALS.

The observed increase in magnetic susceptibility of the putamen in ALS patients is consistent with previous findings. One study reported that the putamen exhibited increased iron accumulation in ALS patients (Acosta-Cabronero et al., 2018). Furthermore, our findings provide quantitative confirmation that the putamen shows significantly increased susceptibility in ALS, suggesting higher iron deposition. By quantifying putamen susceptibility with QSM, we demonstrate a reproducible effect of increased putamen iron levels in ALS. Together with previous results, our findings point to the putamen as a subcortical structure that may be preferentially affected by iron dysregulation in ALS.

These new findings expand the current understanding of iron deposition patterns in the brains of ALS patients, and therefore, they contribute valuable quantitative information on the magnetic susceptibility of the basal nuclei in the context of ALS. Although QSM assessments in ALS are limited, this case study demonstrates their potential for enhancing our understanding of iron dysregulation in ALS and related MNDs. Larger longitudinal studies are needed to validate these results and determine the clinical implications of iron accumulation and overload in ALS.

## Conclusion

QSM is a promising MRI biomarker for evaluating iron levels in neurodegenerative disorders such as ALS. This case report adds to the growing evidence supporting the use of QSM for iron quantification in the brains of ALS patients, with a novel focus on the putamen and GP. QSM is sensitive for detecting iron deposition in both motor and extra-motor areas of ALS patients, providing insights into underlying pathologies. The ability of QSM to quantitatively measure iron content may help enable early diagnosis, disease tracking, and treatment evaluation in ALS. Overall, QSM mapping of iron distribution and overload in the putamen and other subcortical nuclei of ALS patients merits further investigation as a potential imaging marker of disease progression and prognosis.

## Data availability statement

The raw data supporting the conclusions of this article will be made available by the authors, without undue reservation.

## Ethics statement

The studies involving humans were approved by the Ethics Committee of Tehran University of Medical Sciences (Ethical Code: IR.TUMS.MEDICINE.REC.1400.1173). The studies were conducted in accordance with the local legislation and institutional requirements. The participants provided their written informed consent to participate in this study. Written informed consent was obtained from the individual(s) for the publication of any potentially identifiable images or data included in this article. Written informed consent was obtained from the participant/patient(s) for the publication of this case report.

## Author contributions

SG, SM, and FF contributed to the conception and design of the study. SG and SM wrote the initial draft of the manuscript. FF and SB contributed to the clinical analysis. SG, FF, and SB contributed to the revision of the manuscript. All authors read and approved the submitted version.
